# Cardiovascular Outcomes of Semaglutide vs Dulaglutide in Nonobese Type II Diabetes Patients With HFpEF

**DOI:** 10.1016/j.jacadv.2025.101857

**Published:** 2025-06-03

**Authors:** Patrick A. Kwaah, Samuel A. Mensah, Abraham Carboo, Hamza A. Rashid, Emmanuel A. Agyemang, Grace Appah, Smith K. Frimpong

**Affiliations:** a Department of Internal Medicine, Yale School of Medicine, Waterbury, Connecticut, USA; b Department of Internal Medicine, West Virginia University, Morgantown, West Virginia, USA; c Department Internal Medicine, Newark Beth Israel Medical Center, Newark, New Jersey, USA; d Department of Medicine, Geisinger Wyoming Valley Medical Center, Wilkes-Barre, Pennsylvania, USA

**Keywords:** cardiovascular outcomes, dulaglutide, GLP-1 receptor agonists, heart failure with preserved ejection fraction (HFpEF), non-obese, semaglutide, type 2 diabetes mellitus (T2DM)

Glucagon-like peptide-1 receptor agonists (GLP1RAs) are a class of antidiabetic agents known to reduce cardiovascular events in patients with type II diabetes mellitus (T2DM), overweight, and obese individuals.^1^ Recently, there has been increasing evidence of overall cardiovascular benefit of these medications in the management of heart failure with preserved ejection fraction (HFpEF) patients.^2^ Most trials to date have predominantly included obese patients in their sample leaving a critical knowledge gap regarding the efficacy and safety of these agents in nonobese patients, particularly those with HFpEF.^3^ The only study, to the best of our knowledge, that explored GLP1RAs in nonobese T2DM patients with HFpEF showed that GLP1RAs reduce the risk of cardiovascular outcomes such as hospitalization, acute myocardial infarction, and cerebral infarction.^4^ However, whether these benefits extend equally across all GLP1RAs remains unclear. Our study compared the cardiovascular outcomes between semaglutide and dulaglutide in nonobese T2DM patients with HFpEF.

**What is the clinical question being addressed?**

What is the cardiovascular risk difference between semaglutide and dulaglutide in nonobese type II diabetes patients with heart failure with preserved ejection fraction?

**What is the main finding?**

Semaglutide was associated with a lower risk of all-cause mortality and lower rate of diuretic use compared to dulaglutide. There was, however, no difference in hospital readmission.

## Methods

We conducted a retrospective 1:1 propensity-matched cohort analysis of individuals aged >40 years using the TriNetX Analytics Research Network, from its inception in 2013, without applying timeline restrictions. Data were curated on September 19, 2024, with contributions from 58 health care organizations globally. The study adhered to STROBE (STrengthening the Reporting of OBservational studies in Epidemiology) guidelines for observational research. The cohort included individuals with T2DM (International Classification of Diseases-10th Revision-Clinical Modification [ICD 10 CM]: E11) and diastolic heart failure (ICD-10-CM: I50.3). We then excluded patients with systolic heart failure (ICD-10-CM: I50.2) and obesity (ICD-10-CM:E66.0, E66.01, E66.09, E66.1, E66.8, E66.9, Z68.3, Z68.4). Participants were stratified into 2 groups: the semaglutide arm (SA), comprising nonobese individuals with type II diabetes mellitus and HFpEF treated with semaglutide without dulaglutide, and the dulaglutide arm (DA), comprising similar individuals treated with dulaglutide without semaglutide during follow-up. Propensity score matching was applied using 1:1 greedy nearest-neighbor matching algorithm without replacement with a caliper of 0.1 pooled SDs to adjust for potential confounders, including demographics, comorbidities, concurrent medications, and laboratory values (Table 1). Primary outcomes: all-cause mortality, hospital readmission, and diuretic use were identified using corresponding ICD-10 codes postindex date. Risk differences and risk ratios were calculated using modified Poisson regression with statistical significance defined as a 2-sided *P* value <0.05. Sex data were missing for 1.9% of females and 1.6% of males, likely due to incomplete documentation or deidentification protocols. Institutional Review Board approval was not required due to the use of deidentified data.

**Table 1:** Summary of Baseline Characteristics Before and After Propensity Score Matching Presented for Semaglutide and Dulaglutide

	Before Propensity Score Matching			After Propensity Score Matching		
	Semaglutide Group (n = 3,004)	Dulaglutide Group (n = 3,801)	Std Diff.	Semaglutide Group (n = 2,410)	Dulaglutide Group (n = 2,410)	Std Diff.
Age, (y)	70.6 ± 10.2	71.6 ± 10.5	0.091	71.2 ± 10.0	71.2 ± 11.1	0.002
Sex						
Female	49.5%	48.6%	0.017	49.5%	48.9%	0.012
Male	48.6%	50.0%	0.028	48.5%	49.5%	0.019
Race						
White race	62.7%	61.9%	0.019	61.7%	62.0%	0.006
Black or African American	17.3%	19.1%	0.046	18.2%	18.8%	0.015
Hispanic or Latino	7.2%	8.4%	0.044	7.6%	7.7%	0.002
Asian	8.1%	5.9%	0.085	7.6%	7.1%	0.019
Non-Hispanic or Latino	76.6%	69.4%	0.164	74.2%	74.7%	0.011
Comorbidities						
Hypertensive disease	86.4%	85.5%	0.026	86.3%	86.3%	0.002
Ischemic heart diseases	56.5%	53.5%	0.059	55.6%	55.0%	0.013
Cerebrovascular diseases	25.4%	27.8%	0.054	26.7%	27.1%	0.008
Diabetes mellitus	84.6%	83.9%	0.021	84.3%	84.4%	0.002
AKI and CKD	48.9%	53.0%	0.083	50.8	51.6%	0.016
Arterial embolism and thrombosis	1.7%	1.8%	0.012	1.6%	1.7%	0.003
Nicotine dependence	15.1%	14.8%	0.011	15.1%	15.5%	0.010
Alcohol-related disorders	3.3%	4.0%	0.034	3.5%	3.6%	0.007
Lab test						
Hemoglobin A1c	7.8 ± 1.8	8.2 ± 2.0	0.181	7.8 ± 1.8	8.1 ± 1.9	0.164
Cholesterol in LDL	78.3 ± 37.2	77.3 ± 36.3	0.025	78.3 ± 38.0	77.2 ± 34.9	0.031
Body mass index	29.4 ± 5.9	28.7 ± 5.6	0.127	29.2 ± 5.8	28.4 ± 5.5	0.147
Medications						
Antilipemic agents	83.9%	86.6%	0.076	84.7%	84.9%	0.003
ACE inhibitors	41.1%	48.9%	0.155	44.4%	44.4%	0.001
ARB	44.5%	41.3%	0.066	43.3%	43.2%	0.002
Beta-blockers	75.8%	77.2%	0.033	76.2%	76.2%	<0.001
Diuretics	73.2%	74.7%	0.034	73.4%	73.6%	0.003
SGLT2i						
Canagliflozin	3.1%	5.6%	0.119	3.6%	3.8%	0.009
Empagliflozin	24.7%	17.3%	0.182	22.2%	22.5%	0.007
Dapagliflozin	11.5%	8.4%	0.105	10.5%	10.8%	0.008
Ertugliflozin	0.5%	0.4%	0.016	0.5%	0.6%	0.011

Values are mean ± SD or %.

ACE = angiotensin-converting enzyme; AKI = acute kidney injury; ARB = angiotensin II receptor blocker; CKD = chronic kidney disease; Lab = laboratory; LDL = low density lipoprotein; SGLT2i = sodium-glucose cotransporter-2 inhibitor.

## Results

We included 3,004 patients in the SA and 3,810 in the DA. Before propensity score matching, the mean age was 70.6 and 71.6 years in the SA and DA, respectively. Females comprised 49.1% of SA and 48.8% of DA; White individuals made up 62.7% (SA) and 63.0% (DA); Black individuals represented 17.0% (SA) and 17.4% (DA). The mean follow-up was 541 and 518 days in the SA and DA, respectively. Hypertensive diseases were observed in 86.4% of the SA and 85.5% of the DA, while ischemic heart diseases were reported in 56.5% of the SA and 53.5% of the DA. After propensity score matching, 2,410 patients remained in each cohort, with baseline characteristics comparable between groups (Table 1). Semaglutide was associated with a lower risk of all-cause mortality, with 40 cases (1.7%) compared to 72 cases (3.0%) in the DA. This translated to an absolute risk difference of −1.3% (95% CI: −2.2% to −0.5%; *P* = 0.002) and a relative risk (RR) of 0.56 (95% CI: 0.38-0.82) favoring the SA. Similarly, SA had a lower risk of diuretic use compared to DA. The absolute risk difference was −7.9% (95% CI: −12.0% to −3.9%; *P* < 0.001) favoring semaglutide. This corresponded to a RR of 0.71 (95% CI: 0.59-0.85). The risk of hospitalization was similar in both the SA and DA arms, with 116 cases (12.9%) and 114 cases (13.4%), respectively. The absolute risk difference was −0.5% (95% CI: −0.31 to 0.03). This corresponded to a RR of 0.962 (95% CI: 0.76-1.22).

## Discussion

Our study provides insights into the cardiovascular outcomes of semaglutide and dulaglutide in this special population of nonobese T2DM patients with HFpEF, a group that has not been well studied. Semaglutide was associated with a 44% reduction in all-cause mortality and 29% lower risk of diuretic use compared to dulaglutide when assessed as a lifetime risk. However, there was no observed difference in hospital readmission rates between the 2 groups. These findings highlight a potential differential benefit of semaglutide as a preferable therapeutic option in this subgroup.

Although there is considerable literature on the cardiovascular benefits of GLP1RAs, most literature includes obese patients in their population.^1^^,^^3^ The cardiovascular benefits of GLP1RAs have been attributable mainly to its weight reduction, blood pressure lowering, and lipid-lowering effects. They have also been shown to have antiatherogenic and anti-inflammatory properties which also contribute to lowering cardiovascular risk.^5^ Therefore, excluding obesity from our population gives us a unique set of patients allowing us to explore the effects of this class of medication. The observed differential benefit of semaglutide compared to dulaglutide may be due to semaglutide’s higher affinity for GLP-1 receptors and greater potency. Additionally, semaglutide has been shown to reduce inflammation and improve endothelial function, which are critical factors in cardiovascular health.^1^^,^^3^

Limitations of our study include potential undercapture of outcomes outside contributing health systems and lack of data on medication dosage or route of administration. Despite propensity score matching, residual confounding may persist due to the study's retrospective design. The absence of a defined timeline for primary outcomes limits comparability with other studies and ability to assess temporal progression. Endpoints such as body mass index (BMI), cholesterol, and blood pressure at follow-up could not be ascertained. Lastly, ∼30% of patients lacked recorded BMI, though all with BMI ≥30 kg/m^2^ or obesity diagnoses were excluded by design, supporting the focus on nonobese individuals despite incomplete BMI data.

## Conclusions

In summary, our data suggest that nonobese patients with T2DM and HFpEF treated with semaglutide have a lower risk of all-cause mortality and diuretic use compared to those treated with dulaglutide. This finding is clinically relevant, as semaglutide may offer a better treatment option by providing both glucose control and improved cardiovascular outcomes, enabling more informed and personalized care. Future prospective, randomized trials are needed to validate semaglutide cardiovascular benefits in nonobese HFpEF patients and explore the underlying mechanisms of its efficacy.

## Funding support and author disclosures

The authors have reported that they have no relationships relevant to the contents of this paper to disclose.
